# A case report with COVID-19 during perioperative period of lobectomy

**DOI:** 10.1097/MD.0000000000020166

**Published:** 2020-05-29

**Authors:** Peng Han, Fan Li, Peng Cao, Shan Hu, Kangle Kong, Yu Deng, Yukun Zu, Bo Zhao

**Affiliations:** Department of Thoracic Surgery, Tongji Hospital, Tongji Medical College, Huazhong University of Science and Technology, Wuhan, People's Republic of China.

**Keywords:** COVID-19, Perioperative period, SARS-COV-2

## Abstract

**Rationale:**

Currently, COVID-19 has made a significant impact on many countries in the world. However, there have been no reported cases of pulmonary lobectomy with Severe Acute Respiratory Syndrome Coronavirus 2 (SARS-COV-2) infection. We are the first to report such a case.

**Patient concerns:**

We report a 63-year-old Wuhan male patient with smoking history of 40 cigarettes per day for 40 years. He sought medical consultation for right lower lung nodules found by CT scan.

**Diagnoses and interventions:**

The patient's postoperative pathological diagnosis was squamous cell carcinoma of the right lower lung. On the fourth day after the operation, the real-time reverse transcription polymerase chain reaction test showed a positive result. After the operation, we routinely give symptomatic treatments such as anti-infection, nebulization and oxygen inhalation. We also change antibiotics several times depending on the patient's condition.

**Outcomes:**

The patient's condition continued to deteriorate. On the fifth day after surgery, the patient died despite medical treatment.

**Lessons:**

We are the first to report the diagnosis and treatment process of patients with COVID-19 during perioperative period of lobectomy. It provides a case for the postoperative management of such patients.

## 1 Introduction

Lung cancer is the most common malignancy in the world and has a poor prognosis.^[1]^ Lobectomy is the most common radical operation for lung cancer.^[2]^ However, postoperative complications, especially pneumonia, are the leading causes of death in postoperative patients. Pneumonia is one of the most common complications after lung operation, and its incidence in the remaining lung ranges from 2% to 22%.^[3,4]^ The main causes of postoperative pneumonia are aspiration of gastric juice and atelectasis.^[5]^ However, in mid-December 2019, COVID-19 broke out in Wuhan, Hubei Province, China .^[6]^ Currently, the number of confirmed cases is still rising rapidly in several countries. In the presence of the COVID-19 epidemic, the COVID-19 should be taken into account. Surgical trauma and stress responses can lead to weakened immune systems, which also makes patients susceptible to Severe Acute Respiratory Syndrome Coronavirus 2 (SARS-COV-2) and have a higher risk of death after becoming infected. Therefore, on the basis of preventing COVID-19, we should also actively explore the diagnosis and treatment process of patients with COVID-19 after lobectomy. Here, we report a case that provides experience and lessons for the diagnosis and treatment of such patients.

## 2 Case report

**Figure 1:**
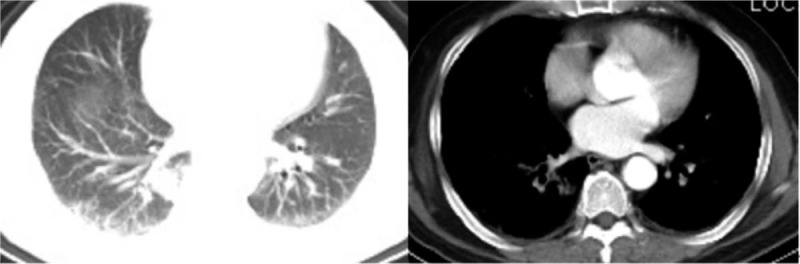
Preoperative chest computed tomography (CT) scan: Right lower lung nodules; interstitial pneumonia; and emphysema.

**Figure 2:**
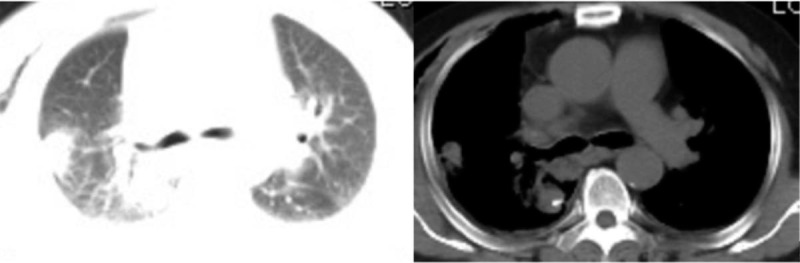
Chest computed tomography (CT) scan the day after surgery: Inflammatory changes in the right lung.

**Figure 3:**
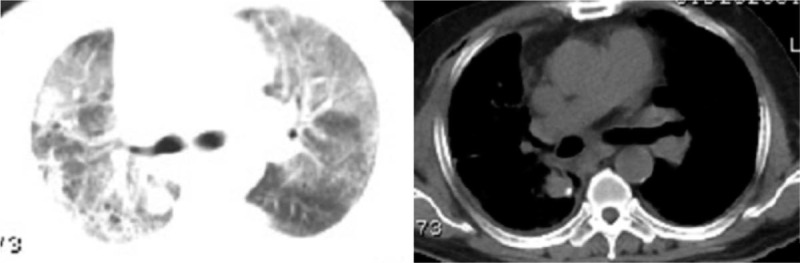
Chest computed tomography (CT) scan the fourth day after surgery: Multiple ground-glass opacities, consolidation, and patchy shadows on both lungs, bilateral pleural effusion with right-side encapsulated hydropneumothorax.

**Figure 4:**
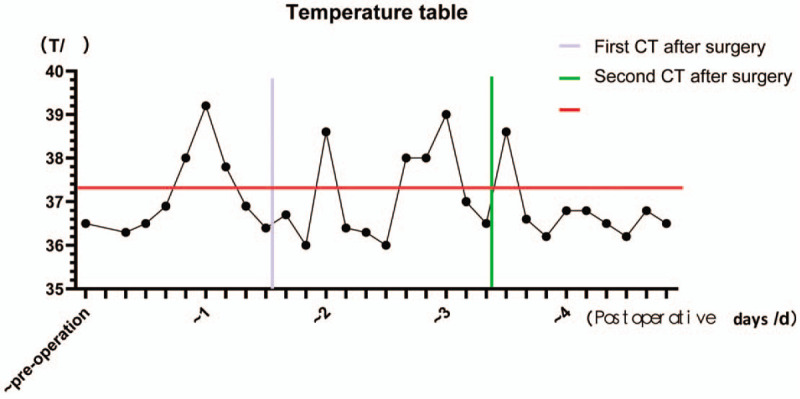
The fact that the patient's body temperature is still high after the first chest computed tomography (CT) scan and the adjustment of antibiotics is also a reminder that we need another chest CT scan.

A 63-year-old male patient was diagnosed with a right lower lung nodule >1 month before admission. The patient usually coughed white foam-like sputum with occasional chest pain. He has no fever, general weakness, and other discomfort symptoms. His previous history includes smoking for 40 years with an average of 40 cigarettes per day, and he has no history of chronic diseases such as hypertension, diabetes, and coronary heart disease. More importantly, the patient denied a history of close contact with the fever patient before admission. A percutaneous lung puncture cytology after admission showed cancer cells, and a chest CT scan before surgery revealed right lower lung nodules, interstitial pneumonia, and emphysema (Fig. 1). Except for patients with gallbladder stones and mild fatty liver, the other routine preoperative tests and examination results were normal. These tests were completed in the hospital. The patient did not go out or had close contact history of Huanan seafood wholesale market and fevering patients during the hospitalization period. The preoperative diagnosis of this patient considered tumorous lesions in the right lower lung with a high probability of malignancy, and the clinical stage was cT2aN0M0. The patient had surgical indications, and no obvious surgical contraindications were seen. After the patient and his family understood the condition and signed the consent form, they requested a single-hole thoracoscopic right lower lobectomy and systemic lymph node dissection. The operation was successfully completed, and the postoperative routine pathological results revealed a middle-differentiated squamous cell carcinoma of the right lower lung without significant catarrhal changes and invasion of the lung membrane. On the first night after the operation, the patient had a sudden high fever of 39.2°C (Fig. 4) with cough and a small amount of purulent sputum, but he said that he did not have significant wheezing and dyspnea. We measured his vital signs and found that the patient's heart rate was 119 beats/min. His auscultation of the lungs showed a few moist rales in the right lung. Therefore, in addition to maintaining the conventional measures such as routine fluid replacement and oxygen inhalation at a flow rate of 5 L/min, we immediately transformed the patient's antibiotics from ceftizoxime sodium + moxifloxacin to ceftizoxime sodium + teicoplanin. The results of laboratory tests (Table 1) the following morning after surgery were as follows: increased white blood cells (16.34 × 10^9^ cells/L), increased neutrophils (14.68 ×10 ^9^ cells/L), and decreased lymphocytes (0.44 × 10^9^ cells/L), mild reduction of hemoglobin and albumin (Table 1). The results of CT scan on the same day suggested inflammatory changes in the right lung (Fig. 2). We immediately gave the patient a bedside fiber bronchoscope treatment, and a small amount of yellow-white sputum was indeed seen in the bronchial cavity of the patient., and the SpO_2_ of the patient can still reach 97% under the stimulation of sputum suction. On the second day after the operation, the patient's body temperature was measured several times during the day (1 time/4 h) and found to be normal. However, the patient's body temperature rose to 38.5°C (Fig. 4) again at 10 pm. So we quickly arranged further laboratory inspections (Table 1): white blood cells increased (13.19 × 10^9^ cells/L), neutrophils increased (11.07 × 10^9^ cells/L), lymphocytes decreased (0.71 × 10^9^ cells/L), high-sensitivity C-reactive protein (CRP) increased (164.9 mg/L), normal procalcitonin (PCT) (0.19), and elevated interleukin 6 (294.60 pg/mL). The results of these laboratory tests were released on the third day after surgery. On the third day after surgery, the patient developed persistent high fever (maximum 39.0°C [Fig. 4]) with chills, but the symptoms of coughing and expectoration were the same as before and no obvious dyspnea was reported. In response to this, we also upgraded the antibiotics of patients from ceftizoxime sodium + teicoplanin to teicoplanin + meropenem while treating them symptomatically. On the same day, we also checked the patient's EB virus DNA, parainfluenza virus 1/2/3 IgM, and influenza A/B virus. IgM, respiratory encapsulation virus IgM, adenovirus IgM, mycoplasma pneumoniae IgM, and chlamydia pneumoniae IgM, among others. But we would not see these results reported until noon on the fourth day after surgery. On the 4th day after surgery, the patient's body temperature showed 38.6°C (Fig. 4) at 6 am, and the temperature measurement every four hours thereafter was within the normal range. However, during the doctor's morning round, the patient said that he felt his dyspnea was slightly worse than before. And we found in the physical examination auscultation that both patients’ lungs showed wet rales, so we urgently applied for a chest CT scan for the patient. CT images showed multiple ground-glass opacities, consolidation, and patchy shadows on both lungs, bilateral pleural effusion with right-side encapsulated hydropneumothorax (Fig. 3); at the same time, the laboratory examination (Table 1) results on the morning of the fourth day after surgery showed that leukocytes increased (7.4 × 10^9^cells/L) and neutrophils increased (6.34 × 10^9^ cells/L), lymphocytes decreased (0.31 × 10^9^ cells/L), lactate dehydrogenase increased (237 U/L), and multiple viral antibody results checked 3 days after surgery showed negative results. We immediately apply for RT-PCR test to further confirm the diagnosis and add gamma globulin to intensive treatment. The patient complained of shortness of breath, chest tightness, and dyspnea after exercise in the afternoon. We adjusted the single channel oxygen supply flow from 5 L/min to 8 L/min, but the above symptoms of the patient continued to progress. After the blood gas analysis showed that the pH was 7.408, PO_2_ 48 mmHg, and PCO_2_ 36.1 mmHg, we adjusted the 8L/min single-channel oxygen supply to 16 L/min dual-channel oxygen supply. But the patient's symptoms still could not be relieved, and the review blood gas showed that the pH was 7.391, PO_2_ 49 mmHg, PCO_2_ 39 mmHg. So we use a noninvasive ventilator (BiPAP mode) to assist patients in breathing. At this time, the patient's oxygen saturation fluctuated between 95% and 98%, and the remaining vital signs were still stable. On the fifth day after surgery, the patient's vital signs were stable and no fever, but he could not leave the ventilator. The results of laboratory tests (Table 1) are as follows: white blood cells increased (6.87 × 10^9^ cells/L), neutrophils increased (6.50 × 10^9^ cells/L), lymphocytes decreased (0.18 × 10^9^ cells/L), lactate dehydrogenase increased (348 U/L), high-sensitivity CRP increased (163.9 mg/L), PCT normal (0.22 ng/mL), erythrocyte sedimentation increased (28 mm/h), interleukin-2 receptor increased (828U/mL), interleukin-6 increased (87.52 pg/mL), interleukin-8 increased (38.6pg/mL), and interleukin-10 increased (14.9 pg/mL). Patients were immediately transferred to the fever ward for further treatment after the RT-PCR test showed a positive result. At the same time, those who were in close contact with the patient were isolated for observation. After transfer, the patient was actively treated with gamma globulin, glucocorticoids, antiviral, anti-infective, and other symptomatic support treatment. With the help of a noninvasive ventilator, his blood oxygen saturation fluctuated between 89% and 100%. Other vital signs are as follows: BP 157/102 mmHg, heart rate 128 to 141 bpm, respiratory rate 36 beats/min. At 9 pm, the patient suffered cardiac arrest and was declared clinically dead despite emergency rescue efforts.

**Table 1:**
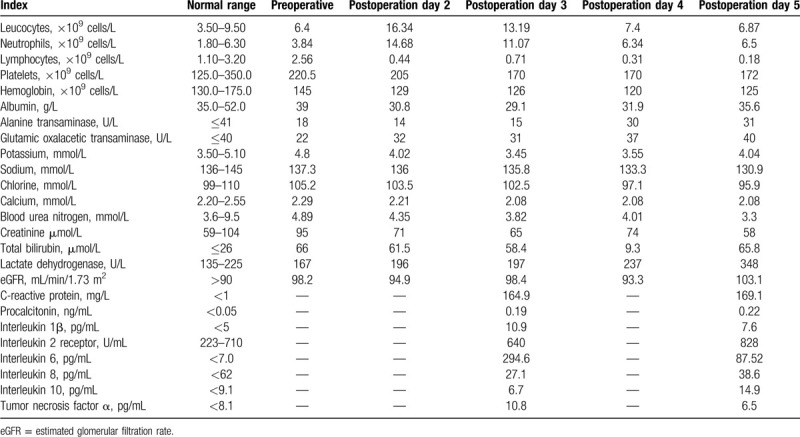
Clinical Laboratory Results.

## 3 Discussion

SARS-COV-2 is a highly infectious enveloped RNA coronavirus (sarbecvirus subgenus, Orthocoronavirinae subfamily).^[7]^ The main mode of transmission is droplet transmission through the respiratory tract, but direct contact transmission and digestive tract transmission cannot be completely ruled out. The incubation period of this virus infection is generally 4 to 8 days.^[8]^ Although the population is generally susceptible, elderly patients with comorbidities have a worse prognosis after infection.^[9,10]^ The most common clinical manifestations of patients with COVID-19 are fever and cough, but a few patients may be accompanied by symptoms such as diarrhea, vomiting, and muscle soreness.^[11,12]^ The patient had a persistent fever from the first day after surgery, accompanied by cough, shortness of breath, shortness of breath, and chest tightness. Although these symptoms are consistent with the typical symptoms of COVID-19, they are also common symptoms in patients after lung surgery.^[4]^ Therefore, it is a huge challenge for us to distinguish between COVID-19 and general pneumonia based on symptomology in patients after lung surgery. At this moment in time, chest CT examination has become our compass. The chest CT image of aspiration pneumonia often present as bronchopneumonia or bronchiolitis with a distinct gravity-dependent distribution.^[13]^ As the most common cause of postoperative pneumonia, gastric contents inhalation can even lead to necrotizing pneumonia and pulmonary abscess with purulent sputum cough and high fever.^[14,15]^ Additionally, bacterial colonization caused by atelectasis is also a common cause of postoperative pneumonia, but chest CT images of this type of pneumonia often show localized changes in unilateral or unilobular lungs. However, the typical chest CT images of COVID-19 are characterized by ground glass opacities, reticular pattern, consolidation, and crazy paving pattern.^[16,17]^ In addition, COVID-19 is often manifested as multiple lobe invasion (46%) and non–gravity-dependent distribution, which is different from aspiration pneumonia and atelectasis.^[8]^ However, the invasion area in chest CT images is associated with disease progression, which is consistent with our case. The chest CT image on the second day after the operation showed consolidation, ground glass opacities, and crazy paving pattern in multiple areas of the right lung with non–gravity-dependent distribution. Moreover, on the fourth day after surgery, the chest CT image showed extensive infiltration and multiple consolidation of both lungs, which was the most typical chest CT image feature of late COVID-19. Furthermore, we also used more powerful antibiotics for this patient, but the patient's condition continued to deteriorate, which did not support the diagnosis of bacterial pneumonia. Indeed, RT-PCR testing is the criterion standard for the diagnosis of COVID-19, mainly owing to its high specificity. However, the study showed that 88% of the 1014 patients had CT findings of pneumonia, whereas only 59% tested positive for RT-PCR.^[18]^ They also found that 97% of the cases confirmed by RT-PCR test had CT findings of pneumonia. Moreover, the turnaround times of RT-PCR test can be lengthy. These results suggest that chest CT scan may be more sensitive than RT-PCR test. Therefore, relying on chest CT images alone, it is reasonable for this patient to be diagnosed as COVID-19. In this case, the patient's condition deteriorated rapidly and eventually led to the death of the patient. After careful consideration, we found that the patient had a history of heavy smoking, interstitial pneumonia, and emphysema, which meant that the lung function of the patient was worse than that of the average person. This finding is consistent with the conclusion that elderly patients with comorbidities have a worse prognosis.^[9,10]^ More importantly, the reduction of the body's immunity caused by surgical trauma and stress response also accelerates the progress and deterioration of the disease.

In conclusion, due to the presence of the COVID-19 epidemic, surgical management should be more cautious. We should conduct more rigorous preoperative screening and actively perform RT-PCR testing when necessary. In patient with COVID-19 after lobectomy, positive CT features can be strongly suggestive of COVID-19, especially when CT images show multiple invasions and non–gravity-dependent distributions. In epidemic area, given that the rapidity and high positive rate of chest CT examination, patients should strictly undergo CT examination from the first day after surgery and repeat the examination according to the changes of the patient's condition. This can not only observe the situation of postoperative pulmonary rehabilitation, but also detect COVID-19 as early as possible. When the chest CT examination indicates a high degree of suspicion, while adjusting the treatment plan, we should quickly isolate the patients individually and conduct RT-PCR assays as soon as possible to confirm the diagnosis. Although the specificity of chest CT scan is significantly lower than that of RT-PCR test, it has higher sensitivity and convenience, which is suitable for the early diagnosis of COVID-19. Moreover, we can also make a comprehensive clinical diagnosis of patients by analyzing the differences between COVID-19 and general pneumonia treatment, and combining the image changes of multiple chest CT examinations.

## Author contributions

**Data curation:** Yu Deng, Fan Li.

**Investigation:** Yukun Zu, Kangle Kong.

**Methodology:** Shan Hu, Peng Cao.

**Revision:** Peng Han, Bo Zhao, Fan Li.

**Supervision:** Bo Zhao, Fan Li.

**Writing – original draft:** Peng Han, Fan Li.
